# Novel Platform for Droplet Detection and Size Measurement Using Microstrip Transmission Lines

**DOI:** 10.3390/s19235216

**Published:** 2019-11-28

**Authors:** Juliana de Novais Schianti, Ariana L. C. Serrano, Daniel Orquiza de Carvalho, Rafael A. Penchel, Julio Mota Pinheiro, Mario R. Gongora-Rubio, Gustavo Pamplona Rehder

**Affiliations:** 1 Institute for Technological Research, São Paulo 05508-901, Brazil; gongoram@ipt.br; 2 Escola Politécnica, Universidade de São Paulo, São Paulo 05508-010, Brazilgprehder@usp.br (J.M.P.); juliomota@usp.br (G.P.R.); 3 Sao Paulo State University (UNESP), São João da Boa Vista SP 13876-750, Brazil; daniel.orquiza@unesp.br (D.O.d.C.); rafael.penchel@unesp.br (R.A.P.)

**Keywords:** emulsion, microfluidics, microstrip transmission line

## Abstract

We propose a novel platform for detecting as well as measuring the size of individual droplets in microfluidic channels using microstrip transmission lines. The most outstanding feature of our platform is that, as opposed to previous related works, its design allows for the droplet to flow in a microfluidic channel fabricated between the top strip and the ground plane of a microstrip transmission line. This provides enhanced interaction of the electromagnetic field with the detected droplets. The proposed design allows us to measure droplet size directly from the phase of the microwave signal, without the need for a resonator. The platform is based on low temperature co-fired ceramic (LTCC), which makes it more compatible with Radiofrequency (RF) and microwave technology than platforms used in previous works. With this platform, we are able to measure droplets as small as 150 µm in radius. It is worth pointing out that our device could also be used for detection, counting and measurement of other microscopic objects.

## 1. Introduction

An emulsion is a liquid dispersion composed of single or multiple droplets suspended in an immiscible continuous phase. These droplets are frequently used as carriers for different types of liquid or solids, including drugs and cells for a breadth of applications. In this way, emulsions have been widely used in the pharmaceutical, chemical and food industries, as well as in material science and biotechnological areas, in order to improve the quality and bioavailability of chemicals, as well as to guarantee protection and encapsulation of drugs providing controlled release [1]. Microfluidic devices have shown to be a promising means for producing emulsion essentially due to the high degree of control over the droplet uniformity in size. By using these devices in parallel, the throughput can be increased significantly, making it comparable to batch production in chemical reactors.

Micro-channels fabricated using different techniques have been successfully used to generate, in a very controlled manner, droplets aimed towards different applications, such as encapsulation of flavors and fragrances [2], vitamins [3] and nanoparticles [4] in the pharmaceutical and food industries. In biotechnology, cells [5,6] and even microorganisms such as algae [7], bacteria and viruses [8] are encapsulated inside droplets for studying these organisms in a controlled environment.

With microfluidic devices, emulsions are produced in a continuous way and with very good control over droplet sizes. These parameters lead to emulsions with a high degree of homogeneity, as well as controlled physical and chemical characteristics. This is combined with other advantages of microfluidic processing, such as low reagent and energy consumption. Droplet characterization is a crucial step for the success of any application concerning emulsions. Information like average size, size homogeneity, composition and concentration, droplet velocity and frequency of droplets formation are key parameters. However, this characterization requires expensive equipment and is conducted sometime after production, usually by using high-speed cameras [9,10,11]. 

Alternative techniques have been proposed in order to have a real time characterization, allowing faster and more precise decisions during the production of droplets for different applications [12,13]. In this respect, many microfluidic devices have been proposed, where micro-channels are integrated with some tool for detection, in configurations that can be capacitive- [14], impedance- [15] or optical-based [16], among others. Methods relying on the electrochemical properties of droplets are a common way to detect many properties. However, not all applications involve materials that have electroactive properties [17,18]. Optical detection has also been applied to characterize droplets. Usually, high-speed camera images are used, and optical fibers combined with lasers and photo-detectors are applied to acquire information [12,13]. These techniques usually require a transparent substrate or the cumbersome integration of optical fibers, which could lead to leakage among other problems.

Recently, techniques that use electromagnetic (EM) waves in the Radiofrequency (RF) and microwave region of the frequency spectrum have been used to perform a label-free detection and measurement of droplet dimensions [19,20]. In these works, microwave resonators [21] with fundamental resonant frequency at 3 GHz were fabricated using microstrip transmission lines on glass substrates. Microfluidic channels were then fabricated on top of the resonators using polydimethylsiloxane (PDMS) and were used to generate droplets. The difference in permittivity of the liquid inside the droplet with respect to the surrounding fluid changes the resonant frequency of the resonator allowing the detection and measurement of the droplets.

In this work we proposed an alternative EM wave-based method where microstrip transmission lines were used in conjunction with microfluidic devices in order to detect as well as measure droplet dimensions directly from the phase shift induced by the local variation of the relative permittivity (ε_r_) due to the presence of such droplets. Being originally designed for RF and microwave applications, low temperature co-fired ceramic (LTCC) substrate (ε_r_ ≃ 7.8) was the platform we chose to fabricate and integrate the microfluidic channels and the microstrip transmission lines used for droplet detection.

## 2. Materials and Methods

### 2.1. Device Structure and Fabrication

**Figure 1:**
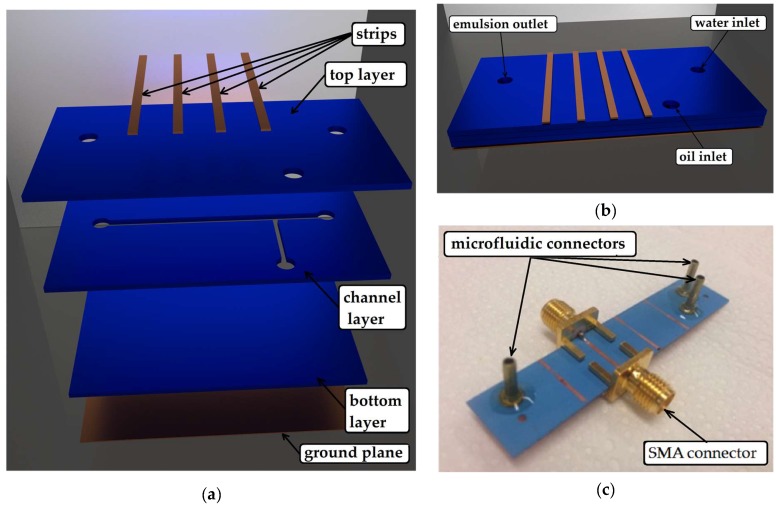
Microfluidic droplet sensor—microstrip transmission line in low temperature co-fired ceramic (LTCC) substrate. (**a**) Expanded view of the different layers of the proposed device, which was composed from the bottom towards the top by: the ground plane of the microstrip transmission line (3 µm-thick copper layer), the 110 µm-thick bottom LTCC sealing layer, the 220 µm-thick LTCC channel layer (actually two layers) with the fluidic channel pattern, the 110 µm-thick top LTCC layer with holes for fluid inflow and outflow and the microstrip strips. The main channel (longer channel) was 5 cm-long, 500 µm-wide and 220 µm-deep. The side channel (shorter channel) associated with oil flow was 3.4 mm-long, 250 µm-wide and 220 µm-deep. The copper strips were 700 µm-wide. Although four strips were patterned on the top surface, only one was used in this work. (**b**) Illustration of the device after fabrication with the two liquid inlets and the emulsion outlet; (**c**) device under test (DUT) with the SubMiniature version A (SMA) connectors for microwave characterization and the microfluidic connectors.

The device used for droplet detection and size measurement in microwave frequencies is illustrated in Figure 1a. The microfluidic device was fabricated using the DuPont Green Tape^TM^ 951P2 LTCC [22,23]. In order to produce single emulsions, a T-junction geometry was designed, as can be seen in the right side of the channel layer in Figure 1a. The width of the longer main microchannel, used for the continuous phase, was 500 µm and that of the shorter orthogonal microchannel, used for the dispersed phase channel, was 250 µm. The ratio of 2:1 in width of the channel associated with the continuous phase flow with respect to the channel associated with dispersed phase flow, allowed us to obtained droplets with different sizes in the range of dimensions around the width values of the aforementioned channels. This was done by varying the continuous and dispersed phases flow values, as will be discussed in the next section. The height of both channels was 220 µm, achieved by using two 110 µm-thick LTCC layers. The use of two LTCC sheets allowed for greater interaction between the electromagnetic mode and the liquids inside the channel, as opposed to using only one LTCC sheet. The microchannels geometry was transferred to the LTCC layers by laser ablation using a LPKF Ultraviolet laser (355 nm, LPKF Protolaser U3, Garbsen, Germany).

After laser ablation, a thermo press machine (MA098/A30, Marconi, Brazil) was used to laminate the aligned ceramic tapes by applying a uniaxial pressure of 11.8 MPa, at a temperature of 70° C. For sealing the microchannels, two 110 µm-thick LTCC layers were pressed on the top and bottom of the channel layer, as shown in Figure 1a. Holes for liquid access were patterned on the top layer by laser ablation. Lamination was followed by sintering, in order to remove the polymeric matrix and solvents from the LTCC layers. After this process, the substrate became rigid. The sintering process was done in a muffle furnace (EDG Equipment, model EDG10P-S, Brazil) in two steps with fixed temperatures: the first one at 350 °C for 30 min, and the second, at 850 °C for 30 min.

The microstrip transmission lines were fabricated by depositing two 10 nm-thick titanium seed layers to promote adherence, followed by two 100 nm-thick copper films on both sides of the LTCC device. This was deposited by the RF sputtering technique after the sintering process described above (Figure 1b). The copper layers were thickened-up to 3 µm by electroplating. Then, four strips were patterned on the top copper layer by photolithography, forming four transmission lines. These transmission lines were designed to have a characteristic impedance of 50 Ω, which resulted in a width of 700 µm in this substrate. SubMiniature version A (SMA) connectors were soldered at both ends of the transmission line for measurements, as shown in Figure 1c. The microfluidic channel crossed underneath the microstrip transmission strip in a 90° angle.

The designed microfluidic device was suitable to produce oil-in-water single emulsion due to the LTCC material having a hydrophilic surface. Using three microfluidic connectors located at each extremity of the T-junction, corn oil was dispersed in a water continuous flow phase (Ultra Pure Water Milli-Q® Direct, Merck KGaK, Darmstadt, Germany) with 5% (v/v) of surfactant (Plantaren 1200, BASF Personal and Nutrition GmbH, Monheim, Germany).

**Figure 2:**
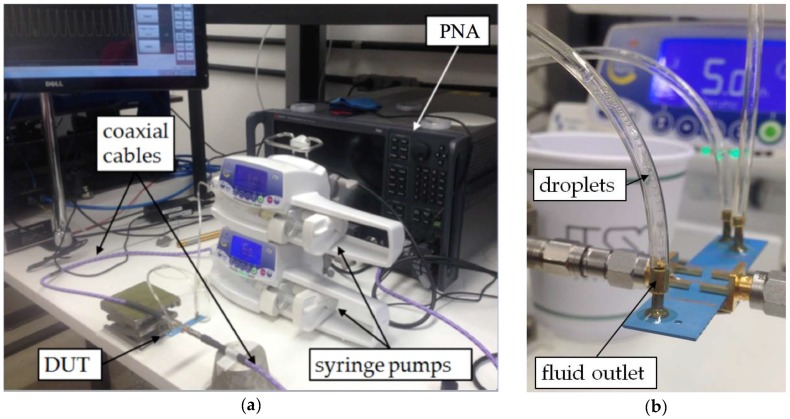
Experimental setup for droplet detection and size measurement: (**a**) complete setup with microfluidic pumps, performance network analyzer (PNA) and device under test (DUT); (**b**) close-up of the microfluidic device.

The parameter used to detect and measure the droplets was the S_21_ scattering parameter. This is a complex quantity the phase of which corresponds to the phase difference between the voltage wave coming out of port 2 (output port) of the device with respect to the phase of the wave entering port 1 (input port). The complete experimental setup is shown in Figure 2a. We used a performance network analyzer (PNA) (N5722B, Keysight, USA), also shown in Figure 2a, to measure the S_21_ parameter of our device as the emulsion crossed paths with the transmission line. The parameter was measured from DC up to 22 GHz, which was the maximum valid frequency limited by the operating range of the SMA connector used in measurement. 

Two syringe pumps (Injectomat MC Agilia, Fresenius Kabi, France) were used to control the liquids flows. In order to obtain different droplet sizes, the water (continuous phase) flow was varied from 7 to 40 mL/h. This also changed the frequency with which the droplets were generated (number of droplets generated per second). In all experiments, the corn oil (dispersed phase) flow was maintained at 0.5 mL/h.

As it can be seen in Figure 1, we conceived the device in such a way that the microfluidic channel was located between the top layer with the strips and the ground plane forming a 90° angle with the microstrip transmission lines. This was done with the purpose of increasing the interaction between the electromagnetic field of the propagating mode of the transmission line and the liquids inside the microfluidic channel, thus improving the sensibility of the sensor. 

The oil droplets were generated in the T-junction of the channel layer in Figure 1a. As the droplet crossed the transmission line, it changed the relative permittivity of the transmission line due to the contrast in relative permittivity between the oil droplet (ε_r_ = 2.2) and water (ε_r_ = 78) surrounding medium, changing its phase constant. This meant that the phase shift acquired when the droplet crossed the channel was different than the phase shift acquired when the channel was filled with the continuous phase (water) only. Once the droplet was detected, counting can be realized by on-line observation or through a simple software analysis of the measured phase shift.

For comparison with the above method using microwave frequencies, the detection of droplets and the measurement of their size were also evaluated by optical methods using a camera and captured images treated using ImageJ.

### 2.2. Electromagnetic Analysis

**Figure 3:**
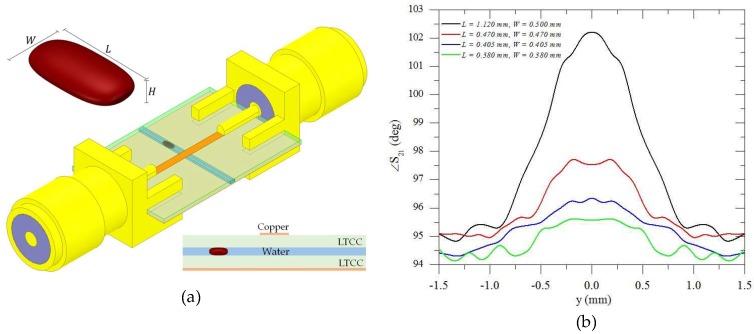
RF microfluidic device for droplet size measurement at 22 GHz: (**a**) geometry of the micro-channel and transmission line with the SMA connectors. (**b**) Simulated S_21_ phase parameter as a function of the droplet position as it crosses the channel.

In order to estimate the electromagnetic performance of the device, a numerical analysis was performed by ANSYS HFSS, which employs the Finite Element Method (FEM). The modeled structure is shown in Figure 3a. The LTCC substrate was 10 mm-wide, 62 mm-long and 0.44 mm-thick. The microchannel had a rectangular cross section of 500 µm × 200 µm. The oil droplet was modeled as an ellipsoid with dimensions L×W×HwithH=220 µm (see Figure 3a) for different *W* and *L* values. *L* and *W* were determined such that the volume remained the same as its spherical form. The two standard SMA connectors inserted at the ends of the transmission line were also taken into account in the EM modeling of the structure. 

Figure 3b shows the EM simulated responses at 22 GHz of the phase of the transmitted signal (/ *S_21_*) obtained for different droplet sizes as a function of the droplet position across the microchannel. The change in magnitude of the transmitted signal does not vary significantly, making it difficult to use for this application. The higher the frequency, the larger the phase shift induced by the change in the relative permittivity, therefore the device can be more sensitive. The frequency of 22 GHz was chosen for all the simulated and measured results for being the highest valid measured frequency. 

As the droplet crossed the microstrip (y = 0.0 mm), the phase varied reaching its maximum at y = 0.0 mm, as expected. From Figure 3b, it is clear that the larger the drop, the greater the variation in the parameter under test. At y=0 the oil droplet was exactly under the transmission line and the maximum deviation from the y=±1.5 position was $\Delta ∠S_{21}\approx 7{}^{\circ}$ for L=1.12 mm and *W* = 0.500 mm. The peak value of the phase of *S_21_* curve could be calibrated to give a direct measurement of the droplet size. 

## 3. Results

**Figure 4:**
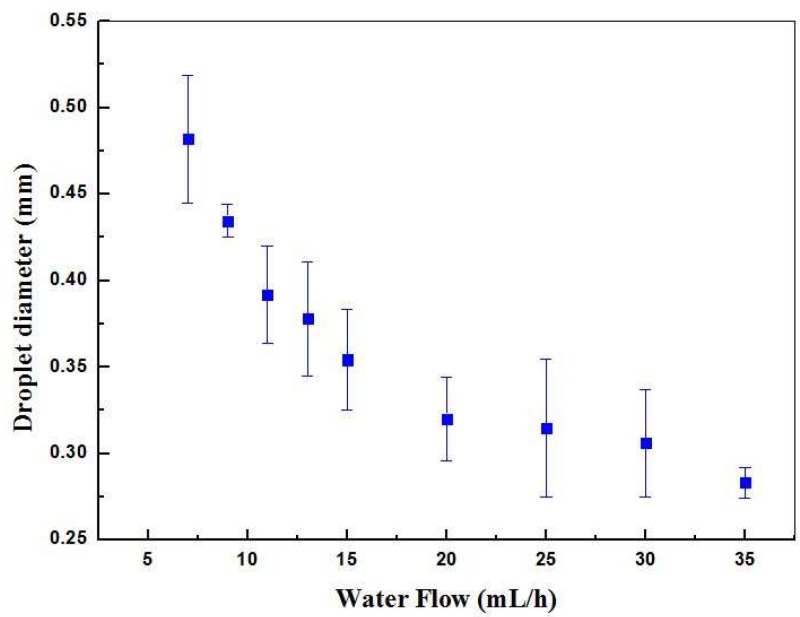
Oil droplet diameter obtained for different water flow values. In all cases, the corn oil (dispersed phase) flow was maintained at 0.5 mL/h.

The optical method was applied to measure the oil droplets diameter using a microscope and image analysis software as a function of the water flow rate, shown in Figure 4. As expected, the droplet size decreased with the increase of the water flow. Droplets with diameter as large as 480 μm were obtained for the lowest water flow value of 7 mL/h. The diameter was largely dependent on the dimensions of the channels that formed the T-junction of the microfluidic device. This could be seen from the fact that the largest droplet diameter (480 μm) corresponded approximately to the width of the microchannel that contains the continuous phase (500 μm). This was expected and rather common for single emulsions produced using microchannels. 

**Figure 5:**
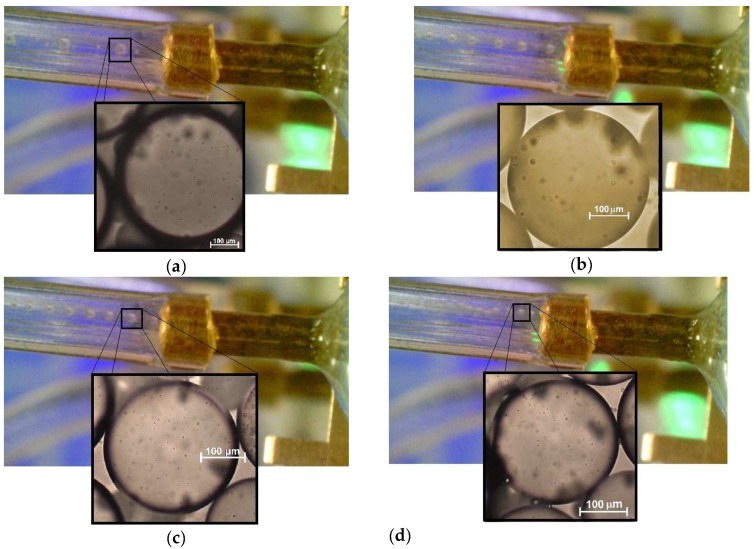
Oil droplets generation rate for four different water flow values: (**a**) 7 mL/h; (**b**) 15 mL/h; (**c**) 20 mL/h; (**d**) 30 mL/h. The insets in each figure show optical microscope images of the corresponding droplets.

As the water flow rate increased to 35 mL/h, the droplets diameter decreased to 310 µm. The maximum flow ratio between phases, i.e., the ratio between water phase and oil phase flows (Q_water_/Q_oil_), was set to 70. It was observed that any flow ratios larger than this value induced the oil phase to be blocked, preventing the formation of droplets in the device. Below this ratio, by increasing the water flow rate, it was also possible to observe an increase in the number of droplets produced per second. Figure 5 illustrates this tendency through images of sequences of droplets with different diameters obtained for four distinct water flow rates (7, 15, 20 and 30 mL/h). The droplet generation rates were 2.6, 5.1, 7.1 and 8.0 droplets/s for continuous phase flow rates of 7, 15, 20 and 30 mL/h, respectively.

**Figure 6:**
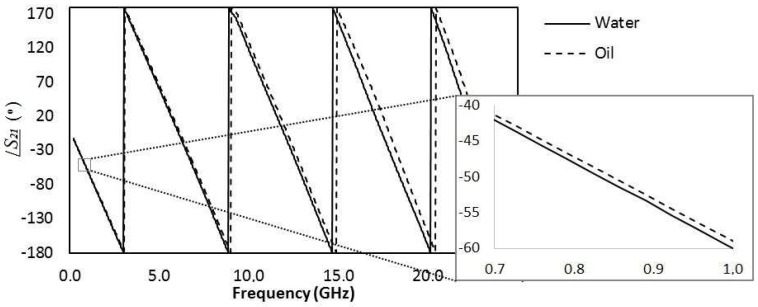
Measured phase response of the transmitted signal for only water (solid line) or only oil (dotted line) flowing through the microchannel. Inset: zoom view of the graph showing phase shift in frequencies lower than 1 GHz.

Considering the method using microwave frequencies, first the transmission line was measured with only water, then only oil flowing through the microchannel. The phase response of the transmitted signal (S_21_) for each case is shown in Figure 6, showing that indeed there was a phase difference when oil or water flows. This figure corroborates the statement explained in Section 2.1 that the higher the frequency, the larger the phase shift between two liquids with different relative permittivities passing through the microchannel. Therefore, we used the higher measured frequency (22 GHz) to provide the information about the droplets, although the phase shift could be detected in lower frequencies such as 1 GHz (inset of Figure 6). Using another frequency would change the “calibration” of the method that correlated the measured phase shift to the dimension of the droplet. Lower frequencies provide smaller phase shift, thus smaller droplets would be harder to measure. Similarly, higher frequencies provide larger phase shift, allowing one to detect and measure even smaller droplets than the ones detected in this article.

**Figure 7:**
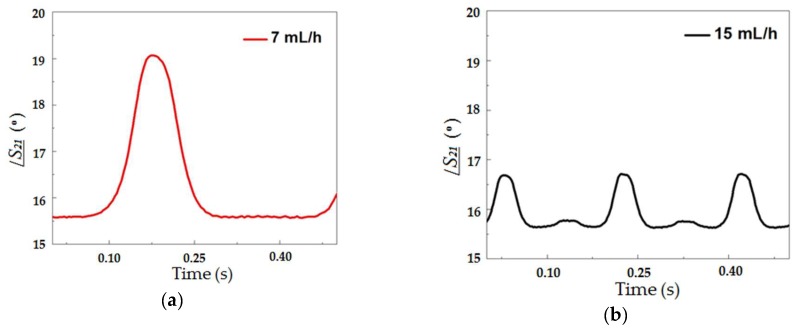

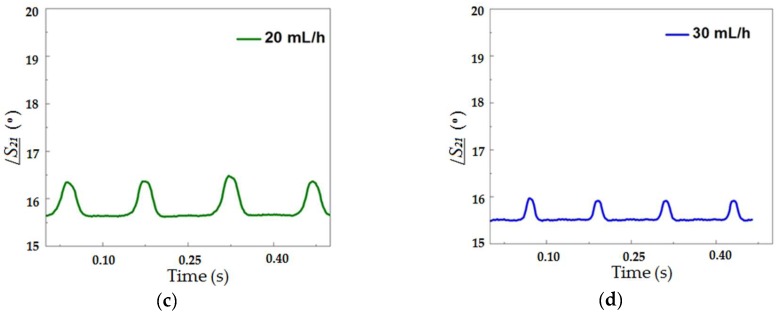
Measured *S_21_* phase as a function of time for different values of continuous phase flow rate: (**a**) 7 mL/h (red), (**b**) 15 mL/h (black), (**c**) 20 mL/h (green) and (**d**) 30 mL/h (blue). The measurements were performed with the performance network analyzer (PNA) setup shown in Figure 2.

Then, the phase of the transmitted signal was measured as a function of time as droplets flowed through the microchannel across the microstrip line. Figure 7 shows this response for a continuous phase flow of 7 (a), 15 (b), 20 (c) and 30 (d) mL/h at 22 GHz. This frequency was chosen for all the results for being the highest valid measured frequency that was limited by the operating range of the connector used. The higher the frequency, the larger the phase shift, although the phase shift could be detected in lower frequencies such as 1 GHz (inset of Figure 6).

As it can be seen in Figure 7, as the droplet crossed the transmission line, the larger the fraction of the droplet underneath the microstrip line, the larger the phase shift measured. This type of curve, aside from allowing one to detect the presence of the droplet at the given position, i.e., the crossing between the fluidic channel and the microstrip, allows for the measurement of the droplet size. The size of the droplets in the emulsion was obtained from the peak value of the curves presented in Figure 7. 

**Figure 8:**
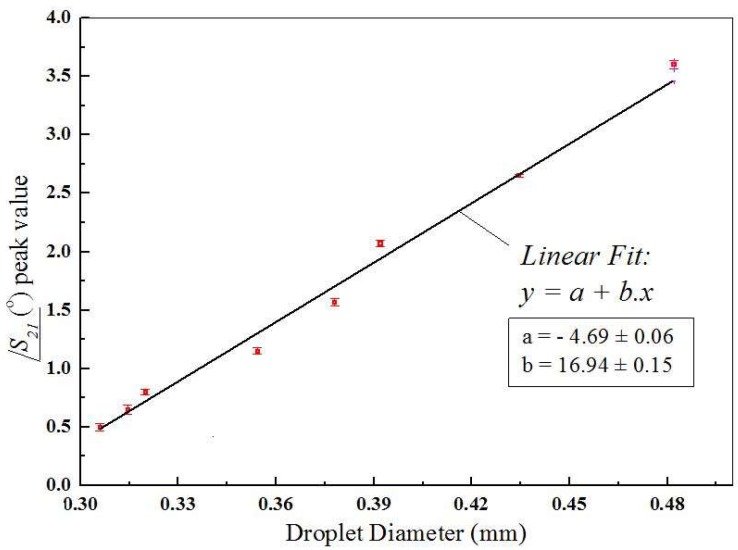
Measured peak of /***S_21_*** plotted as a function of droplet diameter along with the linear regression curve and its parameters.

Each peak in /***S_21_*** shown in Figure 7 corresponds to the presence of a single droplet under the microstrip line. We believe that the difference observed when comparing the maximum phase shifts seen in Figure 3b, obtained with numerical modelling, and those of Figure 7 was due to the fact that the relative permittivity of the liquids are strongly frequency-dependent, and the values used in the simulation $(\varepsilon_{r,water}=81$ and $\varepsilon_{r,oil}=2.2)$ may not correspond to the actual ones at 22GHz, since the permittivities found in the literature are measured at lower frequencies. The main point that can be observed when comparing the curves in Figure 7a–d is that the peak value of /***S_21_*** depends on the continuous phase flow rate and is directly proportional to the droplet diameter. Several curves similar to these were measured and considering the dimensions obtained from the optical method, it resulted in the graph presented in Figure 8, which shows the peaks in /***S_21_*** as a function of the droplet diameter. At the frequency of 22GHz, the peak value of /***S_21_*** induced for droplets with 310 µm in diameter was 0.5°. For droplets with 480 µm diameter this peak was 3.7°. In this graph the linear relation between /***S_21_*** and droplet diameter is clearly observed. This linear relation was observed because the droplet diameters were smaller than the width of the microchannel (500 µm). As the droplets become larger than this value, they will tend to conform to the cross-section dimensions of the microchannel and get elongated in the direction of fluid flow. Though we are not able to generate such larger droplets with our device, these droplets would also be measurable by this device because the width of the curves under the peaks in Figure 7 would become larger as the size of the droplets increase. In that case, a relationship between the peak width and droplet diameter could be found, even if it were not linear as the one seen in Figure 8. In experiments where overlapping droplets are generated, which was not the case in the present work, additional studies would have to be performed in order to find out how the phase and width of curves similar to the ones shown in Figure 7 could be used to distinguish these overlapping droplets.

## 4. Discussion

The microfluidic platform proposed here for droplet detection and size measurement has some key advantages over other platforms used for a similar purpose. A first point that is readily seen in Figure 8 is the linear relationship between the phase of the *S_21_* parameter and the droplet diameter. This was applicable for the range of droplet dimensions studied in this work only. More important than the linear behavior is the fact that the platform proposed here could provide a higher sensibility in terms of phase variation induced by changes in permittivity, when compared to similar platforms [19,20]. The main reason for this is the unique feature, which has to do with the relative position of the microchannel with respect to the microstrip line. Specifically, the microchannel was placed inside the transmission line, between the top metal track and the ground plane. 

**Figure 9:**
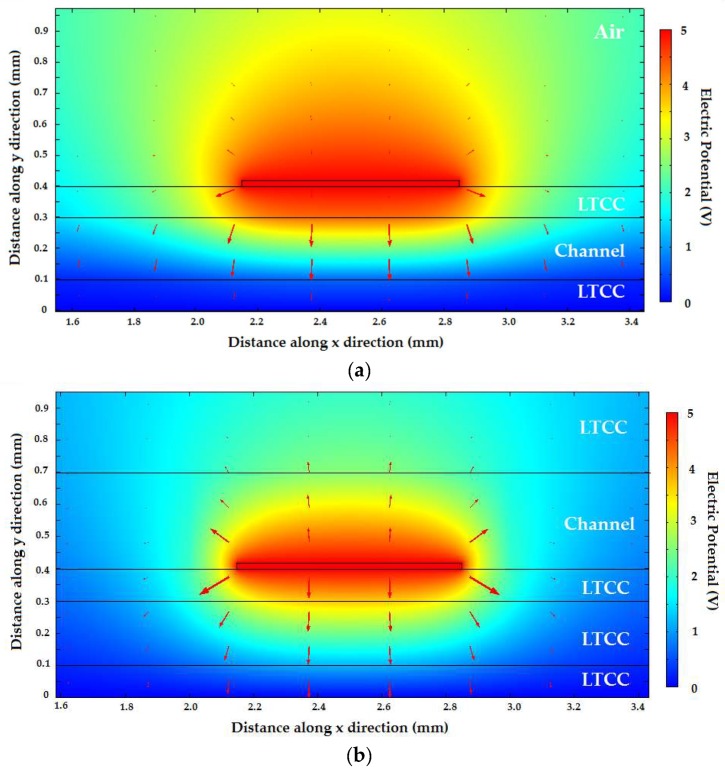
Electric field (red arrows) and electric potential (V) distribution for the Transverse Electromagnetic TEM mode of the microstrip transmission line used in: (**a**) the microfluidic sensor proposed in this work; (**b**) similar sensor with a microstrip line underneath the microfluidic channel.

To obtain a better grasp of how much the phase was affected by variation in permittivity of the liquids surrounding the transmission line, Figure 9a,b shows plots of the electric potential (V) and the electric field (red arrows) of the fundamental mode supported by the transmission lines with two different configurations. These quantities were numerically calculated using Mode Analysis based on the Finite Element Method (FEM). The dimensions and materials used for each layer in the simulation are shown in the Figure. In Figure 9a, the microchannel was placed between the strip and the ground plane. The height of the microchannel was 200 µm, and it was placed in between two 100 µm-thick LTCC sheets. The material above the microstrip was also green ceramic. In Figure 9b, on the other hand, the dielectric substrate between the strip and the ground plane was solid LTCC, and the microchannel was placed on top of the microstrip line. In these figures, the microchannel was filled with oil (ε_r_ = 2.2), but we also analyzed these same structures filled with water, obtaining qualitatively similar results.

It can readily be seen that the intensity of the electric field vector inside the microchannel in Figure 9a is greater than its intensity on top of the transmission line. This is because the channel is in between the metals in this transmission line. By integrating the electric field intensity in the different regions, we calculated the percentage of total Electric Field E-Field of the Transverse Electromagnetic TEM mode which permeated the microchannel in this configuration, which turned out to be 36.7% for the described dimensions. For the configuration of Figure 9b, which is similar to the one used in [19] and [20], the electric field inside the microchannel clearly had a smaller intensity than that of the field underneath the top metal track. For this configuration, only 17% of the field was inside the microchannel in the best case, i.e., using a microchannel with overestimated dimensions of 300 µm in height. 

We also estimated the amount of change in phase constant for two scenarios: when the microchannel was filled with water and when it was filled with oil. For the structure proposed in this work (Figure 9a) the phase constant variation was Δβ = β_water_ − β_oil_ = 378 rad/m. For the structure with the microchannel on top (Figure 9b) the phase constant variation was Δβ = β_water_ − β_oil_ = 259 rad/m. This meant that for the same interaction length of the transmission line and liquid phase, a phase variation 46% higher could be obtained with the microchannel between the strip and the ground plane of the microstrip line.

The intent here was not to directly compare our platform with that used in the aforementioned works, since those works do not describe the dimensions of their devices in detail and also, the substrate material is glass with a top layer which consists of PDMS polymer, as opposed to LTCC. The intent here was to compare the effect of placing the microchannel inside or on top of transmission lines made with the same material platform. A larger fraction of the modal field interacting with the material inside the microchannels translated into a larger induced phase variation caused by change in the permittivity in this region. The key parameter here was the phase constant β. Variations in the permittivity of some portion of the transmission line led to variations in β. The magnitude of the latter can be greater depending on the fraction of the mode field inside the region where the change in permittivity takes place. This is similar to what happens in optical evanescent field-based sensors, where the extent of the evanescent field determines how much β will vary in response to a given change in the refractive index.

The main reason for this phase difference is the fact that, when the electromagnetic mode propagates in a given medium, its phase constant is given by:

(1) $$\beta =\omega \sqrt{\varepsilon \mu },$$

where ω is the angular frequency, μ is the magnetic permeability, which is equal to the magnetic permeability of vacuum ($\mu_{0}=4\pi \times 10^{-7}H/m$) since all media are non-magnetic, and ε is the effective electric permittivity, which depends on the medium surrounding the mode and is very different for water $(\varepsilon_{water}\approx 81\varepsilon_{0}$) and for oil $\left(\varepsilon_{oil}\approx 2.2\varepsilon_{0}\right)$. The permittivity of free space is $\varepsilon_{0}=8.854\times 10^{-12}F/m$.

The phase shift when the electromagnetic mode propagates through a given medium is given by:

(2) φ=βl,

where *l* is the length of interaction of the electromagnetic wave with a given medium in which the mode propagates with a given phase constant β. As seen in Equations (1,2), if an electromagnetic mode, such as the TEM mode of the microstrip line, propagates through media with different ε, such as oil and water, the phase shift is different, which is the reason for the results observed in Figure 3b and in Figure 7.

Aside from the higher sensibility of the structure proposed here, the microchannels were also fabricated on the LTCC substrate, which has many advantages over other materials, such as PDMS soft polymer, which is prone to being attacked by different chemicals and cannot be used for applications which required temperatures higher than 150 °C.

Although the proposed design allowed us to measure droplet size directly from the phase of the microwave signal without the need for a resonator, such a device could lead to a dramatic improvement. This is because resonators allow for small phase variation to be translated into significant amplitude variation. As shown in Reference [20], this could lead to detectors with much larger throughput and even to be able to distinguish the content inside droplets used to encapsulate different substances. Therefore, the next logical step is to use the microstrip line/microchannel configuration proposed here to make devices such as resonators and interferometers. It is very likely that the phase sensibility could translate into a larger variation of the resonant frequencies, although this is beyond the scope of the present work. Finally, by using different microstrip lines to detect the time in which a given droplet crosses these different detectors, it would be possible to determine the speed of the droplets. This could work even for self-propelled droplets such as the metal droplets described in Reference [24]. Although this needs to be studied carefully in future works, the scheme proposed here could also be applied to the detection and measurement of liquid metal droplets. Lastly, since we were able to detect and measure the size of droplets in an emulsion environment, these detectors could be applied in conjunction with switching arrays devised to open specific outlets to enable the separation of droplets with different sizes. This application can be pursued in future works as well.

## 5. Conclusions

We have proposed an integrated device based on a RF microstrip transmission line to detect as well as measure droplet size in single emulsions. The transmission line integrated the microchannel fabricated using standard LTCC technology. It has been demonstrated that there was a linear relationship between the induced phase-shift and the droplet dimension. Moreover, we showed how the proposed design here could lead to better phase sensibility than recent similar works. This was made possible by the use of low temperature co-fired ceramic (LTCC), since sheets of this material can be stacked in such a way that a microfluidic channel can be fabricated in the region between the strips of the transmission line. The device showed great promise for real time measurement and counting of emulsion droplets during production.
